# Die psychische Gesundheit unserer Kinder und Jugendlichen und deren Behandlungsmöglichkeiten im Drei-Länder-Vergleich (Ö, D, CH) unter Berücksichtigung der Veränderungen durch die COVID-19-Pandemie

**DOI:** 10.1007/s40211-022-00438-9

**Published:** 2022-11-09

**Authors:** Kathrin Sevecke, Anna Wenter, Nina Haid-Stecher, Martin Fuchs, Isabel Böge

**Affiliations:** 1 Abteilung für Kinder- und Jugendpsychiatrie, Psychotherapie und Psychosomatik, Tirol Kliniken, Milser Straße 10, 6060 Hall i. T., Österreich; 2 Medizinische Universität Innsbruck, Christoph-Probst-Platz 1, 6020 Innsbruck, Österreich; 3 Universitätsklinikum Graz, Auenbruggerplatz 1, 8036 Graz, Österreich

**Keywords:** Psychische Gesundheit, Kinder, Jugendliche, Epidemiologie, Lebensqualität, Behandlung, Mental health, Children, Adolescents, Epidemiology, Quality of life, Treatment

## Abstract

**Hintergrund und Fragestellung:**

In einer von der Österreichischen Gesellschaft für Kinder- und Jugendpsychiatrie, Psychosomatik und Psychotherapie (ÖGKJP) für die *Neuropsychiatrie *kuratierten Artikelserie erscheinen in einem Schwerpunktheft Arbeiten, die die kinder- und jugendpsychiatrische Versorgungslage zum Thema haben. Der vorliegende Artikel hat sich zum Ziel gesetzt, einen diesbezüglichen Drei-Länder-Vergleich zwischen Österreich, Deutschland und der Schweiz zu machen und Konklusionen für die österreichische kinder- und jugendpsychiatrische Versorgungslandschaft zu ziehen.

**Methode:**

Zunächst werden epidemiologische Eckdaten sowie unterschiedliche Traditionen und Behandlungsphilosophien und deren Niederschlag im öffentlichen Gesundheitssystem der drei deutschsprachigen Länder Österreich, Deutschland und Schweiz vorgestellt. Im Anschluss werden die kinder- und jugendpsychiatrischen Versorgungsdaten von Österreich und Deutschland aufgezeigt und zueinander in Beziehung gestellt.

**Ergebnisse und Schlussfolgerungen:**

In der österreichischen kinder- und jugendpsychiatrischen Versorgung besteht – wenn man die epidemiologische Datenlage, die deutliche Verschlechterung des psychischen Gesundheitszustandes der Kinder und Jugendlichen durch die Pandemie sowie die bisher niedrigen Bettenmessziffern (0,03–0,09) zugrunde legt – dringender Handlungsbedarf. Eine Verbesserung der strukturellen Defizite in der Forschungs- und Versorgungslandschaft, eine deutliche Erhöhung der Kapazitäten im stationären, teilstationären sowie ambulanten Bereich unter Berücksichtigung moderner Strukturen wie des Hometreatments, sind notwendig.

## Abstract (en)

**Background and research question:**

The Austrian Society for Child and Adolescent Psychiatry, Psychosomatics and Psychotherapy (ÖGKJP) is publishing a series of articles in a special issue of *Neuropsychiatrie* dealing with the current provision of care in child and adolescent psychiatry. Aim of this article is to give an overview of provision of care for children and adolescents with mental health problems in Austria, Germany, and Switzerland and then draw conclusions for the Austrian child and adolescent psychiatric care landscape.

**Method:**

First, epidemiologic data as well as different traditions and treatment philosophies and their effect for provision of care in child and adolescent psychiatry in Austria, Germany and Switzerland are presented. Subsequently, data of provided child and adolescent psychiatric care in Austria and Germany are presented and related to each other.

**Results and Conclusions:**

On the basis of the data presented, the current deterioration of mental health in children and adolescents due to the pandemic as well as the deficit of available inpatient beds (0.03–0.09) there is an urgent need for action in Austrian provision of child and adolescent psychiatric care. An improvement of the structural deficits in the child and adolescent psychiatric research and care landscape, a significant increase of capacities for inpatient treatment, day-care as well as outpatient treatment with establishment of modern treatment options such as home treatment is necessary.

## Psychische Gesundheit von Kindern und Jugendlichen

Die europäischen Mitgliedstaaten der Weltgesundheitsorganisation (WHO) wollen eine bessere und bedeutungsvolle Zukunft für die Kinder und Jugendlichen in Europa. Mit ihrer Strategie von der 64. Tagung des Regionalkomitees für Europa 2014 „In Kinder investieren: Strategie der Europäischen Region zur Förderung der Gesundheit von Kindern und Jugendlichen (2015–2020)“ wollen sie eine Basis für gesunde und glückliche Personen schaffen, die einen wichtigen Beitrag zu ihrer eigenen (psychischen) Gesundheit und zu einer starken Gesellschaft leisten [31].

Dennoch gehören weltweit psychische Erkrankungen zu den häufigsten Erkrankungen bei Kindern und Jugendlichen mit einer Punktprävalenz von mindestens 15 % und einer Lebenszeitprävalenz bis zum Erwachsenenalter von bis zu einem Drittel [10]. Zu den am häufigsten vorkommenden psychiatrischen Diagnosen bei Kindern und Jugendlichen zählen Angststörungen (31,9 %), Störungen des Sozialverhaltens (17,7 %), Störungen durch Substanzkonsum (9,9 %), emotionale Störungen (9,0 %), hyperkinetische Störungen (5,4 %) und aggressiv-dissoziale Störungen (4,9 %) [30].

Das Wissen um die Prävalenz und Inzidenz von psychischen Störungen im Kindes- und Jugendalter ist besonders wichtig zur Planung von Behandlungseinrichtungen [4]. An dieser Stelle sollen die wichtigsten epidemiologischen Studien der drei Länder Österreich, Deutschland und Schweiz vergleichend dargestellt und ein Bezug zum jeweiligen Versorgungssystem hergestellt werden. Die kinder- und jugendpsychiatrische Versorgung ist durch eine hohe Komplexität (multiprofessionell), durch unterschiedliche Settings wie ambulant, stationär, teilstationär und aufsuchend bei unterschiedlichen Kostenträgern und unterschiedlich ausgerichteten gesundheitspolitischen Zielen gekennzeichnet. Bei schweren psychiatrischen Erkrankungen sowie bei akuter Eigen- und Fremdgefährdung ist das derzeit vorherrschende Modell eine spezialisierte (akut-)stationäre Behandlung. Jedoch bestehen Kapazitätsengpässe – die sich durch die Auswirkungen der COVID-19-Pandemie deutlich verstärkt haben – aufgrund von fehlenden Behandlungsplätzen und einer geografisch sehr unterschiedlichen Versorgungslage, was im Folgenden aufgezeigt werden soll. Das bedeutet, dass Kinder und Jugendliche immer wieder nicht zeitnah und leitliniengerecht behandelt und ihre psychische Gesundheit nicht entsprechend wiederhergestellt werden kann.

### Epidemiologische Studien zur psychischen Gesundheit in Österreich

Die „Health Behaviour in School-aged Children“-Studie (HBSC) ist die größte Studie zur Kinder- und Jugendgesundheit in Europa. Sie erhebt im Vier-Jahres-Rhythmus Daten zu Gesundheit, Gesundheitsverhalten und Einflussfaktoren auf die Gesundheit von Schüler:innen aus den Sekundarstufen 1 und 2. Die Studie erfasst die Gesundheit und das Gesundheitsverhalten der österreichischen Schüler:innen im Alter von 11, 13, 15 und 17 Jahren. Für die Messung der psychischen Gesundheit wurden die Variablen Lebenszufriedenheit, psychische Beschwerden und emotionales Wohlbefinden herangezogen. Die durchschnittliche Lebenszufriedenheit der österreichischen Schüler:innen vor der COVID-19-Pandemie betrug 7,8 auf einer Skala von 0 bis 10. Der Mittelwert für das emotionale Wohlbefinden lag in der HBSC-Studie 2018 bei 49,5 für Mädchen und 58,0 für Burschen. Ungefähr 24 % der Mädchen und 12 % der Burschen lagen unter dem genannten Schwellenwert und zeigten somit Anzeichen für eine depressive Verstimmung [2].

Darüber hinaus hat sich in Österreich die „Mental Health in Austrian Teenagers“-Studie (MHAT) mit der Epidemiologie im Bereich der psychischen Gesundheit von Kindern und Jugendlichen beschäftigt. 3477 Jugendliche im Alter zwischen zehn und 18 Jahren nahmen daran teil. Ein Großteil des Samples wurde dabei über Schulen (5., 7., 9. und 11. Klasse) in allen Bundesländern rekrutiert. Gleichzeitig wurden aber auch Jugendliche, die den Bildungsbereich verlassen haben und ohne Beschäftigungsverhältnis waren, und Jugendliche in stationärer kinder- und jugendpsychiatrischer Behandlung eingeschlossen.

Zunächst wurden mittels Screeningfragebögen internalisierende und externalisierende Störungen erhoben, bevor in einem weiteren Schritt klinisch-psychologische Interviews zu DSM‑5 Störungen durchgeführt wurden. Die Punktprävalenz für eine psychische Störung lag in der MHAT-Studie bei 23,9 % und es wurde eine Lebenszeitprävalenz von 35,8 % ermittelt. Am häufigsten traten Angststörungen gefolgt von Störungen der neuronalen oder psychischen Entwicklung (inklusive Aufmerksamkeitsdefizit-Hyperaktivitätsstörung und Ticstörungen) und depressiven Störungen auf. Auch ein Geschlechtsunterschied wurde ersichtlich. So fanden die Autor:innen bei Mädchen häufiger internalisierende Störungen, bei Jungen hingegen häufiger Störungen der neuronalen oder psychischen Entwicklung und Verhaltensstörungen.

Nur ca. 50 % der untersuchten Kinder und Jugendlichen mit einer psychischen Störung erhielten auch eine entsprechende Behandlung, was im Umkehrschluss bedeutet, dass knapp 50 % unbehandelt blieben – obwohl sich 18,1 % eine Behandlung wünschten [30]. Bei weiteren 12,7 % wurden zudem nicht die vollständigen Kriterien einer psychischen Störung erfüllt, aber es bestanden deutliche Symptome. Kaum jemand aus dieser Subgruppe gab an irgendeine Form von Unterstützung zu bekommen [16]. Zudem zeigte ein großer Anteil der österreichischen Jugendlichen ein Risiko für eine Essstörung, knapp 31 % der Mädchen und 15 % der Jungen [33].

### Epidemiologische Studien zur psychischen Gesundheit in Deutschland

Auch in Deutschland werden im Rahmen der HBSC-Studie Prävalenzen zum subjektiven Gesundheitszustand, zur Lebenszufriedenheit und zu psychosomatischen Beschwerden von 11-, 13- und 15-jährigen Kindern und Jugendlichen erfasst. Im Jahr 2017/18 schätzten die meisten der *N* = 4347 deutschen Kinder und Jugendlichen ihre Gesundheit und Lebenszufriedenheit positiv ein. Nichtsdestotrotz litt etwa ein Drittel der Mädchen und ein Fünftel der Jungen unter mehreren (multiplen) psychosomatischen Beschwerden. Die Beeinträchtigungen im subjektiven Wohlbefinden zeigten sich vor allem bei Mädchen, älteren Jugendlichen, Jugendlichen mit geringerem familiären Wohlstand sowie bei hoher schulischer Belastung. Eine starke familiäre Unterstützung war hingegen mit einem besseren subjektiven Wohlbefinden assoziiert [14].

Neben der HBSC-Studie gibt es in Deutschland die BELLA-Studie („**BE**fragung zum see**L**ischen Woh**L**befinden und Verh**A**lten“), die Teil der KiGGS-Studie („Studie zur Gesundheit von Kindern und Jugendlichen in Deutschland“) ist, und insbesondere psychische Gesundheit und Wohlbefinden erfasst. Die BELLA-Basiserhebung fand zwischen 2003 und 2006 statt, darauf folgten Welle 1 (2004–2007), Welle 2 (2005–2008), Welle 3 (2009–2011) und Welle 4 (2014–2017). An der Basiserhebung nahmen 2863 Familien mit Kindern und Jugendlichen im Alter von sieben bis 17 Jahren teil. 14,5 % der untersuchten Teilnehmer:innen wiesen nach dem „Strenghts and Difficulties Questionnaire“ (SDQ) mindestens eine psychische Störung auf. Auch hier berichteten weniger als die Hälfte der Kinder eine entsprechende Behandlung zu bekommen [19]. Im Jahr 2020 wurden Ergebnisse eines elfjährigen Beobachtungzeitraums publiziert. 3492 Personen im Alter von sieben bis 31 Jahren nahmen daran teil. Das Wohlbefinden wurde von jüngeren und männlichen Teilnehmern besser eingeschätzt als von älteren und weiblichen. Psychische Probleme in jungen Jahren sagten auch Beeinträchtigungen nach elf Jahren voraus. Laut dieser Studie befand sich nur eines von vier Kindern mit Behandlungsbedarf in psychiatrischer Behandlung [15].

### Epidemiologische Studien zur psychischen Gesundheit in der Schweiz

Die HBSC-Studie in der Schweiz kam zum Ergebnis, dass der Großteil der befragten Schüler:innen über eine hohe Lebenszufriedenheit verfügt. Jungen schätzten ihre Lebensqualität tendenziell etwas höher ein als Mädchen. Während Schweizer Jungen in der HBSC-Studie von 2014 über die verschiedenen Altersgruppen hinweg ein vergleichbares Ausmaß an Stress angaben, nahm bei Schweizer Mädchen der Stress im Jugendalter zu [29].

In der Schweiz gibt es einzelne nicht-repräsentative Studien zu Prävalenzen psychischer Erkrankungen vor allem bei spezifischen Personengruppen, z. B. bei delinquenten Jugendlichen. Es gibt keine aktuellen national repräsentativen Zahlen zur Prävalenz psychischer Störungen bei Kindern und Jugendlichen. Es existieren lediglich Referenzwerte der im Kanton Zürich durchgeführten ZESCAP-Studie („Zurich Epidemiological Study of Child and Adolescent Psychopathology“) von 1994 sowie der darauf aufbauenden Längsschnittstudie ZAPPS („Zürcher Adoleszenten-Psychologie- und Psychopathologie-Studie“), wo zwischen 1994 und 2004/05 Daten erhoben wurden. Die Studien zeigen, dass 22,5 % der Kinder und Jugendlichen im Kanton Zürich in den sechs Monaten vor der Befragung von einer psychischen Erkrankung betroffen waren [27].

### Verschlechterung der psychischen Gesundheit in Zeiten der COVID-19-Pandemie

Mit der COVID-19-Pandemie hat sich die Situation der Kinder und Jugendlichen noch verschärft. Schmidt et al. [20] führten im Frühling 2020 eine repräsentative Studie zur psychischen Gesundheit von Kindern und Jugendlichen (0–19 Jahre) aus Österreich, Deutschland, Liechtenstein und der Schweiz durch. Zwischen 2,2 % und 9,9 % der Kinder und Jugendlichen wiesen emotionale Probleme und Verhaltensprobleme auf. 15,3 bis 43 % berichteten, dass diese Probleme während der Pandemie angestiegen sind. Während bei Kindergartenkindern (1–6 Jahre) der größte Anstieg bei oppositionell-defensivem Verhalten zu verzeichnen war, gaben die Jugendlichen den größten Anstieg bei emotionalen Problemen an [20].

In der „Tiroler COVID-19-Kinderstudie“ gaben die Eltern im Dezember 2021 sowohl für die Kindergarten- als auch die Grundschulkinder mehr internalisierende Probleme und posttraumatische Stresssymptome an als die Eltern zu Beginn der Pandemie im März 2020. Subjektives Bedrohungserleben und ökonomische Schwierigkeiten waren die besten Prädiktoren für eine verschlechterte psychische Gesundheit der Kinder [32]. Der psychischen Gesundheit der österreichischen Jugendlichen (mittleres Alter: 16,3 Jahre, Standardabweichung: 1,4 Jahre) nahmen sich Dale et al. [5] an. Im September bis November 2021 wiesen 62 % der österreichischen Mädchen und 38 % der österreichischen Jungen klinisch relevante depressive Symptome auf. 49 % der Mädchen und 29 % der Jungen hatten klinisch relevante Ängste und 28 % der Mädchen und 17 % der Jungen waren von Schlaflosigkeit betroffen. Die Prävalenz von Suizidgedanken innerhalb der letzten zwei Wochen betrug 47 % bei den Mädchen und 32 % bei den Jungen [5]. In einer „Gesund & Leben“-Umfrage mit österreichischen Schulärzt:innen gaben mehr als 81 % der Ärzt:innen an, dass sich der allgemeine Gesundheitszustand der Schüler:innen in der COVID-19 Pandemie verschlechtert hat. Sämtliche Schulärzt:innen sagten unisono, dass die psychische Belastung aufgrund der COVID-19 Pandemie bei den Schüler:innen zugenommen hat, 78 % meinten sogar „stark“ oder „sehr stark“ [28]. Der Anstieg des Mental-Health-Bedarfes zeigte sich auch an der Abteilung für Kinder- und Jugendpsychiatrie Hall i. T.: Während es im Jahr 2020 keine Veränderung in der Gesamtanzahl der Akutaufnahmen im Vergleich zum Vor-Corona-Jahr 2019 gab, stiegen die Akutaufnahmen im Jahr 2021 um 40 %. Die akute Suizidalität nahm 2021 um 48 % zu [22].

In Deutschland haben Ravens-Sieberer und Kolleg:innen in der Coronazeit die längsschnittliche, repräsentative COPSY-Studie („**CO**rona und **PSY**che“) durchgeführt und die Daten aus der Coronazeit mit den Prä-Corona-Daten der BELLA-Studie verglichen. Vor der COVID-19-Pandemie wiesen 15 % der deutschen Kinder und Jugendlichen eine niedrige gesundheitsbezogene Lebensqualität auf. Dieser Prozentsatz stieg in der Pandemie stark an (Mai/Juni 2020: 40 %; Dezember 2020/Januar 2021: 48 %; September/Oktober 2021: 35 %). Auch die Gesamtprävalenz psychischer Gesundheitsprobleme stieg von 18 % vor der Pandemie auf 29 % zu Erhebungszeitpunkt 1, 31 % zu Erhebungszeitpunkt 2 und 28 % zu Erhebungszeitpunkt 3. Angstzustände und depressive Symptome nahmen zu. Kinder und Jugendliche mit niedriger elterlicher Bildung, beengten Wohnverhältnissen, Migrationshintergrund und psychischen Problemen der Eltern wiesen ein erhöhtes Risiko für Beeinträchtigungen der gesundheitsbezogenen Lebensqualität und der psychischen Gesundheit während der COVID-19 Pandemie auf [18].

Eine Studie mit Schweizer Kinder und Jugendlichen zwischen sieben und 18 Jahren [6] zeigt ein etwas anderes Bild als die österreichischen und deutschen Studien. Während das Wohlbefinden der Schweizer Kinder und Jugendlichen in der ersten Coronawelle im April/Mai 2020 beeinträchtigt war, erholte sich laut Schweizer Daten das Wohlbefinden der Kinder und Jugendlichen im weiteren Verlauf der Pandemie. Die Autor:innen führten die Ergebnisse darauf zurück, dass die Schulen in der Schweiz während der ersten Coronawelle geschlossen und alle Kinder und Jugendlichen im Homeschooling waren, während die Schweizer Schulen während aller folgenden Wellen offen blieben [6].

## Versorgungsstrukturen und unterschiedliche Behandlungsstrategien in Österreich, Deutschland und der Schweiz

### Versorgungssituation Österreich

Eine Einschätzung der Versorgungssituation für Österreich gelingt mit aktuell zur Verfügung stehenden Daten deutlich besser als noch vor einigen Jahren. Einerseits wurden 2017 mit der MHAT-Studie [30] repräsentative epidemiologische Prävalenzdaten für österreichische Kinder und Jugendliche veröffentlicht. Andererseits liegt neben diesen Prävalenzdaten und offiziellen (staatlichen) Messziffern auch eine Publikation [8] vor, die all diese Daten verknüpft und damit wichtige weitere Schlussfolgerungen ermöglicht.

Die GÖG („Gesundheit Österreich GmbH“) veröffentliche zuletzt 2016 das Papier „Integrierte psychosoziale Versorgung von Kindern und Jugendlichen“ [12]. Die stationäre und ambulante medizinische Versorgung von Kindern und Jugendlichen wird im „Österreichischen Strukturplan Gesundheit“ (ÖSG) in der Letztfassung von 2017 geregelt [3]. Der ÖSG gilt als das zentrale Planungsinstrument des österreichischen Gesundheitswesens und ist Bestandteil der laufenden „Zielsteuerung Gesundheit“. Das Dokument wurde zuletzt vom Bundesministerium für Soziales, Gesundheit, Pflege und Konsumentenschutz (BMSGPK) herausgegeben und steht über das „Rechtsinformationssystem des Bundes“ (RIS; ris.bka.gv.at) zum Download zur Verfügung. Für Abteilungen der Kinder- und Jugendpsychiatrie (KJP) sind darin Bedarfsrichtwerte in Form von sogenannten „Bettenmessziffern“ sowie in Form von Erreichbarkeitskriterien festgelegt. Als Bettenmessziffer (BMZ) wird die Zahl von Behandlungsplätzen pro 1000 Einwohner:innen definiert, die zu einer ausreichenden Versorgung notwendig ist. Im Fall der KJP wurden im ÖSG folgende Richtwerte definiert: BMZ von 0,11 (geltend für alle intramuralen Angebote, also vollstationäre sowie tagesstationäre Plätze), Erreichbarkeit innerhalb von 60 min sowie eine minimale Bettenzahl von 30. Im ÖSG ist die real erreichte BMZ für 2018 mit 0,05 angegeben. Damit standen in Österreich 2018 weniger als die Hälfte der für eine ausreichende Versorgung notwendigen voll- bzw. teilstationären Plätze zur Verfügung [3]. Fliedl et al. [8] betonen, dass sich die reale BMZ seither durch einen Ausbau der Kapazitäten auf zuletzt 0,06 erhöht haben dürfte. Zugleich betonen die Autor:innen, dass große regionale Unterschiede bestehen: Die anzustrebende BMZ von 0,11 sei in Vorarlberg und Salzburg nahezu erreicht, in der Steiermark und Wien aber mit 0,04 bzw. 0,05 unter der Hälfte des zu erreichenden Wertes. Die Autor:innen geben an, dass insgesamt österreichweit bei einer BMZ von 0,11 890 voll- bzw. tagesstationäre Plätze zur Verfügung stehen müssten, wovon real im Jahr 2019 lediglich 520 Behandlungsplätze umgesetzt waren [8].

Der stationäre Bereich wird durch ambulante Angebote ergänzt. Diese können einerseits in Form von Ambulanzen bzw. Ambulatorien, andererseits durch niedergelassene Fachärzt:innen erfolgen. Fliedl et al. [8] geben als zu erreichende Versorgungsdichte ein Ambulatorium pro 250.000 Einwohner:innen an, d. h. einen Gesamtbedarf von 36 Ambulatorien für Österreich. Derzeit liegt die Gesamtzahl der verfügbaren Ambulanzen bzw. Ambulatorien in Österreich bei 22. Auch hier zeigen sich große regionale Unterschiede zwischen Regionen ausreichender Versorgung (Salzburg, Vorarlberg) und unterversorgten Bundesländern wie Oberösterreich oder Wien. Die Autor:innen betonen die schwierige Vergleichbarkeit dieser Angebote aufgrund von stark unterschiedlicher Ausstattung und Personalressourcen und damit verbundener Versorgungskapazität [8].

Als dritte Säule der kinder- und jugendpsychiatrischen Versorgung gelten niedergelassene Fachärzt:innen, die in Praxen, teils mit Kassenverträgen, teils als Wahlärzt:innen tätig sind. Hier besteht nach Fliedl et al. [8] österreichweit bevölkerungsbezogen ein Bedarf von 111 vollzeitäquivalenten Kassenstellen zur Minimalversorgung. Von diesen waren 2019 32 Stellen besetzt. Als vierte Säule sind moderne, aufsuchende Behandlungskonzepte wie das Hometreatment, das in Deutschland seit mehreren Jahren erfolgreich und evidenzbasiert durchgeführt wird, unbedingt umzusetzten und die dementsprechenden rechtlichen Rahmenbedingungen zu schaffen.

Zusammenfassend bestehen in Österreich also in allen Versorgungsbereichen (stationär/tagesstationär, Ambulanz, Fachärzt:innen im niedergelassenen Bereich, Hometreatment) nach wie vor große Defizite.

Eine weitere Problematik besteht nach Fliedl et al. [8] in der Personalentwicklung von Fachärzt:innen für KJP bzw. KJPP. Von den Autor:innen wird auf Basis der derzeitigen jährlichen Abschlüsse sowie der zu erwartenden Abgänge durch Pensionierungen eine stagnierende Zahl von Fachärzt:innen für Kinder- und Jugendpsychiatrie (KJP) bzw. Kinder- und Jugendpsychiatrie und Psychotherapeutische Medzin (KJPP) prognostiziert [8]. Das bedeutet, dass eine dünne Personaldecke dem notwendigen Ausbau von Versorgungsstrukturen noch zusätzlich entgegensteht.

Trotz eines fortschreitenden Ausbaus von stationären Kapazitäten sind die Ressourcen diesbezüglich erst in etwa bei der Hälfte der nötigen Plätze angelangt, die vor der Pandemie im ÖSG [3] als ausreichend genannt wurden. Besonders eklatant stellt sich die Lage im Bereich der niedergelassenen Fachärzt:innen dar, wo weniger als ein Drittel der benötigten Praxen zur Verfügung stehen. Eine zu erwartende prekäre Personalsituation verschärft zusätzlich das Versorgungsdefizit.

### Versorgungssituation Deutschland

Wie in einem Artikel von Signorini et al. 2017 [23] beschrieben, hat Deutschland mit 64 Betten pro 100.000 Einwohner:innen unter 18 Jahren die höchste Anzahl an Betten für Kinder und Jugendliche mit psychischen Störungen im Vergleich zu den anderen europäischen Ländern. Das statistische Bundesamt in Deutschland veröffentlicht jährlich die Grunddaten der Krankenhäuser. Demnach waren 2020 an 143 Fachkrankenhäusern oder Fachabteilungen für KJP 6699 Betten für Kinder und Jugendliche mit psychischen Störungen vorbehalten. Eine Auslastung der aufgestellten Betten bestand zu 78,2 %. Insgesamt 56.943 Kinder und Jugendliche wurden 2020 stationär aufgenommen und mit einer durchschnittlichen Verweildauer von 33,7 Tagen stationär behandelt.

Auch an tagesklinischen Plätzen war in Deutschland ein deutlicher Zuwachs in den letzten Jahrzehnten zu verzeichnen (2004: 1454 Plätze – 2020 : 3895 Plätze = +168 %). Dies führte allerdings nicht im erwarteten Ausmaß zur Entlastung vollstationärer Kapazitäten, stattdessen scheinen durch die teilstationären Betten offenbar insgesamt mehr Kinder und Jugendliche mit psychischen Auffälligkeiten erreicht zu werden. Im Jahr 2020 wurden in 157 Tageskliniken (TK) 24.632 Patient:innen teilstationär aufgenommen [25].

Ambulant werden einerseits in aller Regel an den Krankenhäusern niederfrequente diagnostische bzw. vor- und nachstationäre Behandlungstermine in den Institutsambulanzen abgehalten, andererseits gewährleisten niedergelassene Kinder- und Jugendpsychiater:innen, approbierte Kinder- und Jugendlichenpsycholog:innen sowie Kinder- und Jugendlichenpsychotherapeut:innen die ambulante Versorgung von Kindern und Jugendlichen mit psychischen Störungen.

Im Jahr 2014 waren 1018 Kinder- und Jugendpsychiater:innen deutschlandweit tätig [7]. In den Krankenhäusern arbeiteten 2019 1070 Kinder- und Jugendpsychiater:innen, davon waren 205 leitende Ärzt:innen für KJP, 498 arbeiteten als Oberärzt:innen. 367 Assistenzärzt:innen befanden sich in Ausbildung zur/zum Kinder- und Jugendpsychiater:in [25].

Trotz dieser sehr guten Ausstattung an stationären aber auch ambulanten Behandlungsplätzen zeigte sich in der BELLA-Studie, dass weniger als die Hälfte der behandlungsbedürftigen Kinder und Jugendlichen sich in einer kinder- und jugendpsychiatrischen/-psychotherapeutischen Behandlung befanden [11].

Als das „Gesetz zur Weiterentwicklung der Versorgung und Vergütung für psychiatrische und psychosomatische Leistungen“ (Psych VVG) am 01.01.2017 in Kraft trat, wurde damit zudem die Grundlage geschaffen, um alternativ zur stationären Behandlung intensives Hometreatment als Krankenhausbehandlung für psychisch kranke Kinder und Jugendliche regelhaft anzubieten. Es wurde hierfür der § 39 des SGB V um den Zusatz ergänzt: „Versicherte haben Anspruch auf stationsäquivalente Behandlung durch ein nach § 108 zugelassenes Krankenhaus, wenn die Aufnahme oder die Behandlung im häuslichen Umfeld nach Prüfung durch das Krankenhaus erforderlich ist, weil das Behandlungsziel nicht durch teilstationäre, vor- und nachstationäre oder ambulante Behandlung einschließlich häuslicher Krankenpflege erreicht werden kann. Die stationsäquivalente Behandlung umfasst dabei eine psychiatrische Behandlung im häuslichen Umfeld durch mobile ärztlich geleitete multiprofessionelle Behandlungsteams. Sie entspricht hinsichtlich der Inhalte sowie der Flexibilität und Komplexität der Behandlung einer vollstationären Behandlung.“ Seitdem sind in Deutschland inzwischen an ca. zehn Standorten zusätzlich mobile Behandlungsteams entstanden, Tendenz steigend, mit jeweils 5–10 Behandlungsplätzen, welche multiprofessionell unter der Leitung eines/einer Fachärzt:in für KJP aufsuchende Behandlung im häuslichen Umfeld der psychisch erkrankten Kinder und Jugendlichen unter Einbezug der Eltern anbieten [1].

### Versorgungssituation Schweiz

Das schweizerische Gesundheitsobservatorium (Obsan) berichtete 2017 in einem Dossier, dass im Bereich der psychiatrischen und psychotherapeutischen Versorgung von Kindern und Jugendlichen zwischen 2006 und 2016 wenig empirische Literatur publiziert wurde. Mehrere Berichte zur Versorgungssituation wiesen deutlich auf eine Unterversorgung in der KJPP, sowohl für den institutionellen als auch für den privaten Sektor, hin.

Laut einer Hochrechnung gab es über 29.000 ambulante institutionelle Behandlungen. 67 % der Institutionen führten eine Warteliste, wobei bei 10 % der Institutionen über drei Monate auf einen Behandlungstermin gewartet werden musste. Nur vier von 23 Kantonen gaben an, über eine gute Abdeckung der stationären KJP zu verfügen und zehn Kantone gaben an, über eine gute Abdeckung ambulanter KJP zu verfügen. Vier Kantone berichteten von einem großen Mangel an Psychiater:innen (inklusive Kinder- und Jugendpsychiater:innen) [29].

2020 gab es in der Schweiz insgesamt 717 Kinder- und Jugendpsychiater:innen [9]. Außerhalb des institutionellen Settings bieten niedergelassene Kinder- und Jugendpsychiater:innen und psychologische Psychotherapeut:innen psychiatrische und psychotherapeutische Versorgung für Kinder und Jugendliche an. Alle stationär angestellten Psychiater:innen im KJP-Bereich berichteten, dass sie mangels Kapazität Patient:innen abweisen mussten. Bei den ambulanten und intermediären Angeboten waren es 64 % der Ärzt:innen, die von Überweisungen aus Kapazitätsmangel berichteten. Die Stellen von Fachärzt:innen im KJP-Bereich bleiben in der Schweiz teilweise über drei Monate vakant. Dieser Mangel an Kinder- und Jugendpsychiater:innen zeigte sich unabhängig von Region und Setting [29].

## Vergleich der Versorgungssituation in Österreich und Deutschland

### Vergleich der Bettenmessziffern in Österreich und Deutschland

In Tab. 1 ist die aktuelle Versorgungssituation in Österreich mit Stand 06/22 entsprechend einer von der Österreichischen Gesellschaft für Kinder- und Jugendpsychiatrie, Psychosomatik und Psychotherapie (ÖGKJP) durchgeführten Befragung dargestellt [21]. Hierbei sind nur die Betten an kinderpsychiatrischen Primariaten berücksichtigt – psychosomatische Betten an pädiatrischen Primariaten fehlen.

**Table 1:** 

Bundesländer	Versorgungsregionen	Einwohner:innen pro Bundesland^a^ [24]	Einwohner:innen 0–18 J. pro Bundesland^a^ [24]	% 0–18 J. pro Bundesland^a^ [24]	Anzahl Betten (KJP & Psychosomatik^b^) [21]	TK-Plätze [21]	BMZ pro Bundesland^c^
Burgenland	Burgenland	297.583	50.096	16,83	0 (ab 2027: 20)	Keine	–
Kärnten	Klagenfurt	564.513	96.166	17,04	31	8	0,05
Niederösterreich	Hinterbrühl (NÖ und nördl. Burgenland)	1.698.796	3035	18,49	30	16 (ab Okt. 2022: 20)	0,05
	Mauer bei Amstetten				30	6	
	Tulln				20 (ab 2024: +4 Eltern/Kind)	10	
Oberösterreich	Linz, 2 Standorte	1.505.140	288.195	19,15	54	22	0,04
	Grieskirchen				27	5	
Salzburg	Salzburg	562.606	104.398	18,56	30	10	0,07
	Schwarzach im Pongau				12	Keine	
Steiermark	Graz (Steiermark und südl. Burgenland)	1.252.922	214.059	17,08	33 (ab 2023: 53)	14 (ab Okt. 2022: 20)	0,03
Tirol	Hall i. T.	764.102	140.457	18,38	37	6	0,05
	Innsbruck					5	
Vorarlberg	Bregenz	401.674	81.086	20,19	36	8	0,09
	Rankweil					4	
Wien	Medizinische Universität Wien	1.931.593	350.695	18,16	30	8	0,05
	Wien, Floridsdorf				24	6	
	Wien, Klinik Hietzing				43	0	
	Wien, Psychosoziales Netzwerk				0	10 (–12)	
*Österreich*	*–*	*8.978.929*	*1.639.187*	*18,26*	*437*	*138*	*0,05*

*NÖ* Niederösterreich, *OÖ* Oberösterreich

^a^Per 01.01.2022

^b^Ausschl. KJP-psychosomatische Betten, d. h. ohne psychosomatische Betten an der Pädiatrie

^c^Ohne TK-Plätze

Die BMZ in Österreich variiert zwischen den einzelnen Versorgungsregionen. In Vorarlberg liegt die BMZ bei 0,09, während sie in der Steiermark (inklusive südliches Burgenland) bei lediglich 0,03 liegt. Demgegenüber stehen die Empfehlungen im ÖSG, der eine BMZ von 0,07–0,13 fordert [3, 21]. Mit Stand 06/2022 hatte keine der österreichischen Versorgungsregionen, die im ÖSG empfohlenen Minimalwerte erreicht.

Tab. 2 gibt parallel zur österreichischen Versorgungssituation einen Überblick über die Versorgungssituation in den deutschen Bundesländern. Auch in Deutschland variiert die BMZ zwischen den einzelnen Bundesländern (0,06 bis 0,16). Im Vergleich zu der BMZ für Österreich von 0,05 (siehe Tab. 1) ist die BMZ für Deutschland mit 0,08 (siehe Tab. 2) fast doppelt so hoch.

**Table 2:** 

Bundesländer	Einwohner:innen^a^ [26]	Einwohner:innen 0–18 J^a^ [26]	% 0–18 J^a^ [26]	Anzahl Betten (KJP, 2020) [25]	BMZ pro Bundesland^b^
Baden-Württemberg	11.124.642	2.011.109	18,08	647	0,06
Bayern	13.176.989	2.322.315	17,62	756	0,06
Berlin	3.677.472	647.080	17,60	267	0,07
Brandenburg	2.537.868	429.276	16,91	241	0,09
Bremen	676.463	119.921	17,73	50	0,07
Hamburg	1.853.935	332.008	17,91	197	0,11
Hessen	6.295.017	1.128.553	17,93	557	0,09
Mecklenburg-Vorpommern	1.611.160	260.753	16,18	211	0,13
Niedersachsen	8.027.031	1.429.897	17,81	740	0,09
Nordrhein-Westfalen	17.924.591	3.213.978	17,93	1273	0,07
Rheinland-Pfalz	4.106.485	715.908	17,43	274	0,07
Saarland	982.348	156.178	15,90	60	0,06
Sachsen	4.043.002	682.489	16,88	371	0,09
Sachsen-Anhalt	2.169.253	341.793	15,76	354	0,16
Schleswig-Holstein	2.922.005	503.799	17,24	403	0,14
Thüringen	2.108.863	341.152	16,18	298	0,14
*Deutschland*	*83.237.124*	*14.636.209*	*17,58*	*6699*	*0,08*

^a^Per 31.12.2021

^b^Ohne TK-Plätze

Um das Versorgungsangebot insgesamt vergleichen zu können, muss man neben der jeweiligen BMZ noch das ambulante, tagesklinische und Hometreatment-Angebot berücksichtigen.

## Diskussion

Psychische Störungen bei Kindern und Jugendlichen werden auch als sogenannte „neue Morbidität“ bezeichnet, die eine hohe Krankheitslast zeigen und zur Chronifizierung neigen. Insbesondere früh auftretende Entwicklungs- und psychische Störungen weisen transgenerationale Effekte auf und gehören zu den stärksten Prädiktoren für lebenslange Teilhabebeeinträchtigungen. In Zeiten von Pandemie und weiterer gesellschaftlicher Belastungen wie dem Ukraine-Krieg hat sich die Situation der psychischen Gesundheit unserer Kinder und Jugendlichen weiter verschärft.

In der vorliegenden Arbeit wurden die psychische Gesundheit bzw. die Prävalenz psychischer Störungen im Kindes- und Jugendalter vor und während der Pandemie anhand der vorhandenen Studien in Österreich, Deutschland und der Schweiz beleuchtet. In einem nächsten Schritt wurden die bestehenden Versorgungsstrukturen in den drei Ländern dargestellt sowie die Versorgungssituation in Österreich und Deutschland verglichen. Ziel der vorliegenden Arbeit war es, den kinder- und jugendpsychiatrischen Behandlungsbedarf in Österreich aufzuzeigen.

Die vorliegende Arbeit zeigt, dass die kinder- und jugendpsychiatrische Versorgungssituation in Österreich – auch im Vergleich zu den beiden deutschsprachigen Nachbarländern Deutschland und Schweiz (siehe z. B. BMZ von 0,05 in Österreich versus BMZ von 0,08 in Deutschland) – prekär ist. Auch im ambulanten und teilstationären Setting sowie im Hometreatment schneidet Österreich im Vergleich deutlich schlechter ab. Zurzeit ist aufgrund des Fehlens in vielen Strukturen eine qualitativ hochwertige und differenzierte Versorgung punktuell, nicht aber strukturell, möglich. Die Abstimmung der Versorgungsstrukturen auf Versorgungsebene muss daher in Zukunft Grundlage der flächendeckenden Versorgungsplanung des ÖSG sein. Hier müssen die Prinzipien der Wohnortnähe, der Qualität und der Übergänge zwischen den Versorgungseinrichtungen gleichermaßen berücksichtigt werden (best point of care).

Ausgehend von den Ergebnissen der vorliegenden Arbeit scheint es den Autor:innen dringend notwendig in Österreich in die psychische Gesundheit von Kindern und Jugendlichen zu investieren, zumal die Pandemie die schon vorher bestehenden psychischen Auffälligkeiten und Versorgungsdefizite wie durch ein Brennglas drastisch verschärft hat. An folgenden Punkten sollte angesetzt werden:

- A) Kinder- und jugendpsychiatrische Versorgung
  2020 hat der Marburger Bund als perspektivische Strategie ein Positionspapier zur Zukunft der deutschen Krankenhausversorgung aus ärztlicher Sicht veröffentlicht, wo eine bedarfsgerechte Versorgung der Bevölkerung mit leistungsfähigen Krankenhäusern gefordert wird [13]. Für die österreichische kinder- und jugendpsychiatrische Versorgungssituation fordern die Autor:innen ausgehend von der vorliegenden Analyse:
  - Ausbau der kassenfinanzierten Niederlassungen von Fachärzt:innen für KJP
  - Ausbau der kassenfinanzierten Psychotherapie für Kinder und Jugendliche
  - Ausbau der tagesklinischen Versorgung
  - Anhebung der BMZ auf die im ÖSG [3] geforderten Bedarfsrichtwerte
  - Implementierung neuer Versorgungsmodelle wie Hometreatment als stationsersetzende, wohnortnahe Behandlungsform
  - Partizipation und Mitbestimmung der Patient:innen und ihrer Familien bei der Auswahl des Settings
  - Aufbau kooperativer Strukturen mit benachbarten Fächern (Pädiatrie, Psychiatrie)
  - Aufbau kooperativer Strukturen mit den Systemen der Kinder- und Jugendhilfe, der Justiz sowie des Bildungssystems

Darüber hinaus sollte an den Schulen (B) und in der Forschung (C) in die psychische Gesundheit unserer Kinder und Jugendlichen investiert werden.

- B) Primärprävention an Schulen
  - Integration des Themas psychische Gesundheit in die Schule, Schulinhalte in Richtung psychische Gesundheit erweitern
  - Schulsupportpersonal für psychische Gesundheit: Ausbau von Schulpsychologie, Schulpsychotherapie, Schulsozialarbeiter:innen und Vertrauenslehrer:innen
- C) Monitoring und Forschung zur psychischen Gesundheit der österreichischen Kinder und Jugendlichen
  Monitoring und Forschung sind notwendig, denn gute Versorgung braucht verlässliche Zahlen. So hat auch *Public Health Schweiz* aufgerufen, dass bessere Gesundheitsdaten für ein effizienteres Gesundheitswesen dringlich sind. Präzise Versorgungsdaten sind unabdingbar, um das Behandlungsangebot den Bedürfnissen der Kinder und Jugendlichen anzupassen. Studien und Daten sollen helfen, Maßnahmen in Prävention und Behandlung zu planen [17].

## Schlussfolgerung

Die vorliegende Arbeit hat gezeigt, dass – wenn man die epidemiologische Datenlage, die deutliche Verschlechterung des psychischen Gesundheitszustandes der Kinder und Jugendlichen durch die Pandemie sowie die im Vergleich sehr niedrigen Bettenmessziffern zugrunde legt – in der österreichischen kinder- und jugendpsychiatrischen Versorgung dringender Handlungsbedarf besteht. Eine zeitnahe, abgestufte Versorgung und leitliniengerechte Behandlung muss sichergestellt sein. Hierfür sind eine Verbesserung der strukturellen Defizite in der Versorgungslandschaft, eine deutliche Erhöhung der Kapazitäten im stationären, teilstationären sowie ambulanten Bereich und ein Ausbau des Hometreatments unbedingt notwendig.

## References

[1] Boege I, Schepker R, Grupp D (2020). Kinder- und jugendpsychiatrische stationsäquivalente Behandlung (StäB): Therapieoption – für alle oder für wenige?. Z Kinder Jugendpsychiatr Psychother.

[2] Bundesministerium für Arbeit, Soziales, Gesundheit und Konsumentenschutz. Die psychische Gesundheit österreichischer Schülerinnen und Schüler. HBSC-Factsheet 01: Ergebnisse der HBSC-Studie. 2018. https://www.sozialministerium.at/Themen/Gesundheit/Kinder--und-Jugendgesundheit/HBSC.html. Zugegriffen: 13. Sept. 2022.

[3] Bundesministerium für Soziales, Gesundheit, Pflege und Konsumentenschutz. Österreichischer Strukturplan Gesundheit. 2017. https://goeg.at/sites/goeg.at/files/inline-files/%C3%96SG_2017_-_Textband%2C_Stand_01.10.2021.pdf. Zugegriffen: 13. Sept. 2022.

[4] Costello EJ, Burns BJ, Angold А (1993). How can epidemiology improve mental health services for children and adolescents?. J Am Acad Child Adolesc Psychiatry.

[5] Dale R, Jesser A, Pieh C (2022). Mental health burden of high school students, and suggestions for psychosocial support, 1.5 years into the COVID-19 pandemic in Austria. Eur Child Adolesc Psychiatry.

[6] Ehrler M, Hagmann CF, Stoeckli A (2022). Mental sequelae of the COVID-19 pandemic in children with and without complex medical histories and their parents: well-being prior to the outbreak and at four time-points throughout 2020 and 2021. Eur Child Adolesc Psychiatry.

[7] Fegert JM, Kölch M, Krüger U. Sachbericht zum Projekt: Versorgung psychisch kranker Kinder und Jugendlicher in Deutschland – Bestandsaufnahme und Bedarfsanalyse. 2017. https://www.bundesgesundheitsministerium.de/fileadmin/Dateien/3_Downloads/K/Kindergesundheit/Versorgung_psychisch_kranke_Kinder_u_Jugendliche_Abschlussbericht.pdf. Zugegriffen: 14. Sept. 2022.

[8] Fliedl R, Ecker B, Karwautz A (2020). Kinder- und jugendpsychiatrische Versorgung 2019 in Österreich – Stufen der Versorgung, Ist-Stand und Ausblick. Neuropsychiatrie.

[9] Foederatio Medicorum Helveticorum Generalsekretariat. Berufstätige Ärzteschaft nach Hauptfachgebiet und Kanton; 2020. 2020. https://www.fmh.ch/themen/aerztestatistik/fmh-aerztestatistik.cfm#i145526. Zugegriffen: 14. Sept. 2022.

[10] Fuchs M, Karwautz A (2017). Epidemiologie psychischer Störungen bei Kindern und Jugendlichen. Eine narrative Übersichtsarbeit unter Berücksichtigung österreichischer Daten. Neuropsychiatrie.

[11] Hintzpeter B, Klasen F, Schön G (2015). Mental health care use among children and adolescents in Germany: results of the longitudinal BELLA study. Eur Child Adolesc Psychiatry.

[12] Kern D, Ladurner J. Integrierte psychosoziale Versorgung von Kindern und Jugendlichen. 2016. https://www.sozialministerium.at/Themen/Gesundheit/Gesundheitssystem/Gesundheitssystem-und-Qualitaetssicherung/Planung-und-spezielle-Versorgungsbereiche/Psychosoziale-Versorgung-von-Kindern-und-Jugendlichen.html. Zugegriffen: 13. Sept. 2022.

[13] Marburger Bund Bundesverband. Zukunft der Krankenhausversorgung aus ärztlicher Sicht. Positionspapier des Marburger Bundes. 2020. https://www.marburger-bund.de/sites/default/files/files/2020-09/MB-Positionspapier_Zukunft_der_Krankenhausversorgung-final.pdf. Zugegriffen: 13. Sept. 2022.

[14] Moor I, Winter K, Bilz L (2020). Die Health Behaviour in School-aged Children (HBSC) 2017/18 – Methodik der Kinder- und Jugendgesundheitsstudie der Weltgesundheitsorganisation. J Health Monit.

[15] Otto C, Reiss F, Voss C (2021). Mental health and well-being from childhood to adulthood: design, methods and results of the 11-year follow-up of the BELLA study. Eur Child Adolesc Psychiatry.

[16] Philipp J, Zeiler M, Waldherr K (2018). Prevalence of emotional and behavioral problems and subthreshold psychiatric disorders in Austrian adolescents and the need for prevention. Soc Psychiatry Psychiatr Epidemiol.

[17] Public Health Schweiz. Manifest. Bessere Gesundheitsdaten für ein effizienteres Gesundheitswesen. 2013. https://public-health.ch/de/aktivit%C3%A4ten/mani/. Zugegriffen: 14. Sept. 2022.

[18] Ravens-Sieberer U, Erhart M, Devine J (2022). Child and adolescent mental health during the COVID-19 pandemic: results of the three-wave longitudinal COPSY study. SSRN Journal.

[19] Ravens-Sieberer U, Wille N, Erhart M (2008). Prevalence of mental health problems among children and adolescents in Germany: results of the BELLA study within the national health interview and examination survey. Eur Child Adolesc Psychiatry.

[20] Schmidt SJ, Barblan LP, Lory IA (2021). Age-related effects of the COVID-19 pandemic on mental health of children and adolescents. Eur J Psychotraumatol.

[21] Sevecke K, Wenter A, Böge I. Stationäre Versorgungskapazitäten in der Kinder- und Jugendpsychiatrie - wer hat Platz? Neuropsychiatrie. 2022. 10.1007/s40211-022-00443-y PMC9643980 36348224

[22] Sevecke K, Wenter A, Schickl M (2022). Stationäre Versorgungskapazitäten in der Kinder- und Jugendpsychiatrie – Zunahme der Akutaufnahmen während der COVID-19 Pandemie?. Neuropsychiatrie.

[23] Signorini G, Singh SP, Boricevic-Marsanic V (2017). Architecture and functioning of child and adolescent mental health services: a 28-country survey in Europe. Lancet Psychiatry.

[24] Statistik Austria. Bevölkerung zu Jahresbeginn nach Bundesland, Alter, Geschlecht sowie österreichischer/ausländischer Staatsangehörigkeit 2002 bis 2022. 2022. https://www.statistik.at/statistiken/bevoelkerung-und-soziales/bevoelkerung/bevoelkerungsstand/bevoelkerung-nach-alter/geschlecht. Zugegriffen: 13. Sept. 2022.

[25] Statistisches Bundesamt. Grunddaten der Krankenhäuser – 2020. 2022. https://www.destatis.de/DE/Themen/Gesellschaft-Umwelt/Gesundheit/Krankenhaeuser/Publikationen/Downloads-Krankenhaeuser/grunddaten-krankenhaeuser-2120611207004.pdf?__blob=publicationFile. Zugegriffen: 14. Sept. 2022.

[26] Statistisches Bundesamt. Tabelle. Bevölkerung: Bundesländer, Stichtag, Altersjahre. 2022. https://www-genesis.destatis.de/genesis/online?operation=abruftabelleBearbeitenlevelindex=1levelid=1662117397287auswahloperation=abruftabelleAuspraegungAuswaehlenauswahlverzeichnis=ordnungsstrukturauswahlziel=werteabrufcode=12411-0012auswahltext=werteabruf=starten#abreadcrumb. Zugegriffen: 2. Sept. 2022.

[27] Steinhausen HC, Winkler Metzke C, Meier M (1998). Prevalence of child and adolescent psychiatric disorders: the Zürich epidemiological study. Acta Psychiatr Scand.

[28] Strobl R (2022). Schulärzte schlagen Alarm. Gesund Leben Österreich.

[29] von Wyl A, Chew Howard E, Bohleber L, et al. Psychische Gesundheit und Krankheit von Kindern und Jugendlichen in der Schweiz: Versorgung und Epidemiologie. Eine systematische Zusammenstellung empirischer Berichte von 2006 bis 2016. 2017. https://www.obsan.admin.ch/sites/default/files/2021-08/obsan_dossier_62_2.pdf. Zugegriffen: 12. Sept. 2022.

[30] Wagner G, Zeiler M, Waldherr K (2017). Mental health problems in Austrian adolescents: a nationwide, two-stage epidemiological study applying DSM-5 criteria. Eur Child Adolesc Psychiatry.

[31] Weltgesundheitsorganisation. In Kinder investieren: Strategie der Europäischen Region zur Förderung der Gesundheit von Kindern und Jugendlichen (2015–2020). 2014. https://www.euro.who.int/__data/assets/pdf_file/0004/253768/64wd12g_InvestCAHstrategy_140440.pdf. Zugegriffen: 13. Sept. 2022.

[32] Wenter A, Schickl M, Sevecke K (2022). Children’s mental health during the first two years of the COVID-19 pandemic: burden, risk factors and posttraumatic growth—a mixed-methods parents’ perspective. Front Psychol.

[33] Zeiler M, Waldherr K, Philipp J (2016). Prevalence of eating disorder risk and associations with health-related quality of life: results from a large school-based population screening. Eur Eat Disord Rev.

